# Supplementary material for: Predicting visual function by interpreting a neuronal wiring diagram
Source: Nature. 2024 Oct 2;634(8032):113–23. doi: 10.1038/s41586-024-07953-5 (PMC11446822; doi:10.1038/s41586-024-07953-5)
Supplement: Supplementary file 6 — CRF and ERF predictions for individual TmY4 and TmY9 cells. Analogous to Supplementary Data 3, but for TmY target types. Shown are the top four monosynaptic pathways, the strongest pathway passing through each of the top ten intermediary types (ranking from Extended Data Fig. 7), and the trisynaptic pathway Tm1–TmY–Dm3–TmY (see the section entitled Prediction of spatial normalization). [file 41586_2024_7953_MOESM6_ESM.zip › DataS4/TmY9q/720575940620886369.pdf]

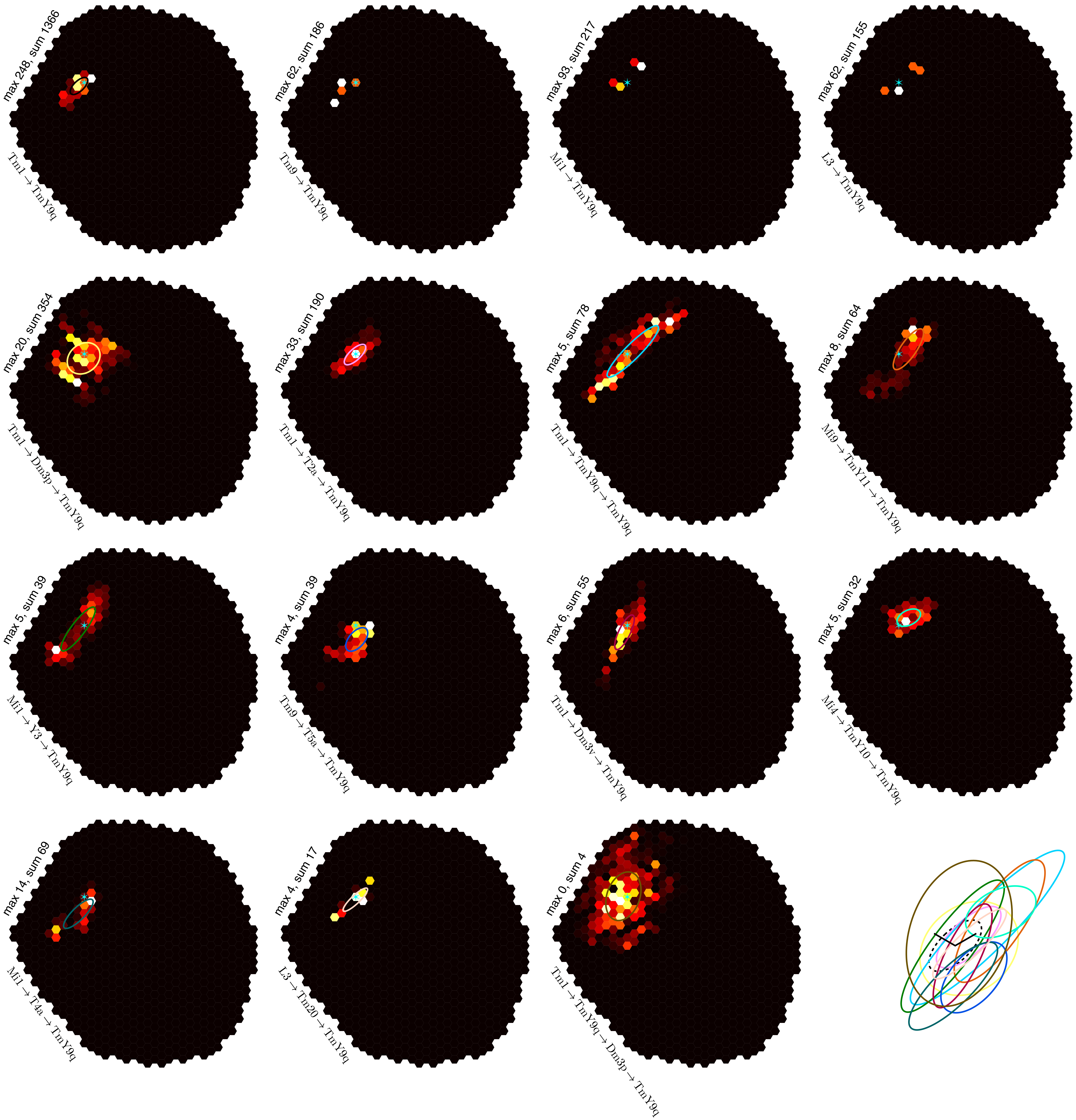
max 248, sum 1366
Tm1 → TmY9q
max 62, sum 186
Tm9 → TmY9q
max 93, sum 217
Mi1 → TmY9q
max 62, sum 155
L3 → TmY9q
max 20, sum 354
Tm1 → Dm3p → TmY9q
max 33, sum 190
Tm1 → T2a → TmY9q
max 5, sum 78
Tm1 → TmY9q → TmY9q
max 8, sum 64
Mi9 → TmY11 → TmY9q
max 5, sum 39
Mi1 → Y3 → TmY9q
max 4, sum 39
Tm9 → T5a → TmY9q
max 6, sum 55
Tm1 → Dm3v → TmY9q
max 5, sum 32
Mi4 → TmY10 → TmY9q
max 14, sum 69
Mi1 → T4a → TmY9q
max 4, sum 17
L3 → Tm20 → TmY9q
max 0, sum 4
Tm1 → TmY9q → Dm3p → TmY9q
